# Comment on Dalboni da Rocha et al. Artificial Intelligence for Neuroimaging in Pediatric Cancer. *Cancers* 2025, *17*, 622

**DOI:** 10.3390/cancers17111776

**Published:** 2025-05-26

**Authors:** Elif Keles, Mehmet Numan Colakoglu, Max Bengtsson

**Affiliations:** 1 Department of Radiology, Feinberg School of Medicine, Northwestern University, Chicago, IL 60611, USA; maxbengtsson2025@u.northwestern.edu; 2 Department of Radiology, Ankara Bilkent City Hospital, 06800 Ankara, Türkiye; mehmetnumancolakoglu@gmail.com; 3 McCormick School of Engineering, Northwestern University, Evanston, IL 60208, USA

As researchers actively working in the field of pediatric neuroimaging and artificial intelligence, we were pleased to see your recent review, Artificial Intelligence for Neuroimaging in Pediatric Cancer, by Rocha et al. [1], which reflects the growing interest and rapid progress in this area. Reviews like this are critical for synthesizing knowledge and guiding future research directions.

However, we felt compelled to write this letter to clarify several points in Table 1, as some of the reported segmentation results may inadvertently misrepresent the original studies or oversimplify important distinctions—particularly regarding model evaluation metrics, dataset characteristics, and segmentation targets. These discrepancies are not limited to a single line but involve several studies where important methodological details—such as whether the reported Dice scores reflect whole-tumor or subregion segmentation, or whether metrics like Pearson’s correlation were used—were either unclear or omitted. These inconsistencies, while subtle, can lead to confusion in how readers interpret and compare model performance across studies.

Our intention is to support clarity, consistency, and transparency in the evaluation of AI models in pediatric brain tumor segmentation—especially as the field moves toward clinical integration. We respectfully offer the following clarifications for improved accuracy and transparency.

Examples of clarification:

- Boyd et al. [2] reported a whole-tumor Dice score of 0.88 for low-grade gliomas, based on external datasets from CBTN (n = 60) and DFCI/BCH (n = 100), using T2-weighted MRI. The model employed a stepwise transfer learning strategy, starting from BRATS 2021 adult glioma weights (n = 1251) and fine-tuned on CBTN data (n = 124). This methodological context is essential for interpreting the reported performance.
- Mulvany et al. [3] presented Dice scores ranging from 0.657 to 0.967, reflecting different tumor subregions. The 0.931 Dice score corresponds specifically to whole-tumor segmentation on the PED BraTS 2024 validation dataset (n = 91), with a training set of n = 261 cases.
- Vossough et al. [4] reported a Dice score of 0.9 for whole-tumor segmentation on an internal test set (n = 293) and a Pearson correlation coefficient of 0.98, which reflects volume agreement rather than spatial overlap. These are distinct metrics and should not be used interchangeably.
- Our study [5] reported a Dice score of 0.642 as an average across tumor subregions (enhancing, non-enhancing, cystic, and edema). The whole-tumor Dice score was 0.681 on CBTN (n = 30 LGG cases) and 0.866 on the held-out PED BraTS 2024 dataset (n = 26). This distinction between whole-tumor and subregion metrics is important when making cross-study comparisons.

To support **consistent and accurate comparisons of AI segmentation models**, distinguishing between **whole-tumor Dice scores and subregion Dice scores** in tables and discussions would improve **clarity and transparency** [6,7].

We appreciate your consideration of this clarification. Providing **clear segmentation performance metrics, dataset details, and model specifications** is important for advancing AI applications in pediatric neuroimaging [7,8].

To facilitate accurate and meaningful comparisons across segmentation models, we suggest that future tables and summaries distinguish between

- Whole-tumor and subregion Dice scores,
- Spatial overlap (Dice) versus volume-based (e.g., Pearson r) metrics;
- Internal versus external validation cohorts, with appropriate dataset specifications.

We hope these clarifications are helpful and contribute to the broader goal of advancing transparency and reproducibility in pediatric neuroimaging AI research.
